# Dietary Fatty Acids and Predementia Syndromes

**DOI:** 10.1100/tsw.2009.82

**Published:** 2009-08-11

**Authors:** Vincenzo Solfrizzi, Vincenza Frisardi, Cristiano Capurso, Alessia D' Introno, Anna M. Colacicco, Gianluigi Vendemiale, Antonio Capurso, Francesco Panza

**Affiliations:** ^1^ Department of Geriatrics, Center for Aging Brain, Memory Unit, University of Bari, Italy; ^2^ Department of Geriatrics, University of Foggia, Italy; ^3^ Internal Medicine Unit, IRCSS Casa Sollievo Dalla Sofferenza, San Giovanni Rotondo, Italy

**Keywords:** MUFA, PUFA, fatty acids, predementia syndromes, dementia, Alzheimer’s disease, vascular dementia, mild cognitive impairment, age-related cognitive decline

## Abstract

An increasing body of epidemiological evidence suggests that elevated saturated fatty acids (SFA) could have negative effects on age-related cognitive decline (ARCD). Furthermore, a reduction of risk for cognitive decline and mild cognitive impairment (MCI) has been found in population samples with elevated fish consumption, and high intake of monounsaturated fatty acids (MUFA) and polyunsaturated fatty acids (PUFA), particularly n-3 PUFA. However, recent findings from clinical trials with n-3 PUFA supplementation showed efficacy on depressive symptoms in non–Vapolipoprotein E (APOE) ε4 carriers, and on cognitive symptoms only in very mild Alzheimer's disease (AD) subgroups, MCI patients, and cognitively unimpaired non-APOE ε4 carriers. These data, together with epidemiological evidence, support the idea that n-3 PUFA may play a role in maintaining adequate cognitive functioning in predementia syndromes, but not when the AD process has already taken over. Therefore, at present, no definitive dietary recommendations on fish and unsaturated fatty acids consumption, or lower intake of saturated fat, in relation to the risk for dementia and cognitive decline are possible.

## INTRODUCTION

Clinical and epidemiological research has focused on the identification of risk factors that may be modified in predementia syndromes, at a preclinical or early clinical stage of dementing disorders. In recent years, in an effort to identify clinical targets of potential therapeutic agents for Alzheimer's disease (AD), people with mild cognitive impairment (MCI) have been enrolled in trials with drugs that were tested in patients with AD[1]. However, previous studies have shown that not all MCI subjects have predementia AD[2]. The umbrella term "predementia syndromes" includes the transitional phase between mild nondisabling cognitive decline and disabling dementia, an ambiguous diagnostic period during which it is unclear whether mild cognitive deficits predict incipient dementia or not. In fact, the clinical label identifies all conditions with age-related deficits in cognitive function reported in the literature, including a mild stage of cognitive impairment based on a normality model and pathological conditions considered predictive of early stages of dementia[2,3]. Such predementia syndromes have been defined for AD and partly for vascular dementia (VaD), but have not yet been operationalized for other specific forms of dementia.

## Definition of Predementia Syndromes

The term "predementia syndromes" includes different conditions and among them, MCI is, at present, the most widely used term to indicate nondemented, aged persons with no significant disability, and a mild memory or cognitive impairment that cannot be explained by any recognized medical or psychiatric condition[2,3]. At present, the term mild cognitive impairment and its acronym MCI have frequently been used in studies on the preclinical phases of dementia, although with differing and inconsistent definitions[4,5,6,7]. There is now ample evidence that MCI is often a pathology-based condition with a high rate of progression to AD[2,3]. Therefore, MCI has also been identified as the predementia syndrome for AD. The more recently proposed multiple subtypes of MCI were intended to reflect the heterogeneity of different types of dementia. Actually, there are at least three different subclassifications of MCI according to its cognitive features[8], clinical presentation[7], and probable etiology[9]. MCI definitions can be broadly classified as amnestic (aMCI) and nonamnestic (naMCI). A critical review was recently made in Stockholm and then in Montreal, in order to define a new consensus on MCI[7]. Modification of Petersen's criteria[6] was proposed during the conference in Montreal. Lastly, the European Consortium on Alzheimer's Disease (EADC) working group on MCI very recently proposed a novel diagnostic procedure with different stages, combining neuropsychological evaluation and family interview to detect MCI at the earliest possible stage[10]. Furthermore, different diagnostic criteria have been proposed for other predementia syndromes, and the terms age-related cognitive decline (ARCD)[11] and aging-associated cognitive decline (AACD)[12] have recently been proposed to distinguish individuals with mild cognitive disorders associated with aging, and nonpathologically based from noncognitively unimpaired individuals.

The causes of predementia syndromes and dementia are unknown at present. However, some studies have suggested that these conditions may be prevented[13,14]. The role of the diet in cognitive decline has not been investigated extensively, with a few data available on the role of macronutrient intake in the pathogenesis of predementia and dementia syndromes[13,14]. Since several dietary factors affect the risk of cardiovascular disease, it can be assumed that they also influence the risk of dementia[15]. Some recent studies have suggested that dietary fatty acids may play a role in the development of cognitive decline associated with aging or dementia[16]. This concept is further supported by recent evidence that certain diets have been associated with a lower incidence of AD. In fact, antioxidants, dietary fatty acids, and micronutrients appear to have a role, and evidence is at least suggestive that diets rich in fruits and vegetables and other dietary approaches may permit a beneficial effect on the risk of dementia[13,14].

Fatty acids can be categorized briefly into saturated fatty acids (SFA) and unsaturated fatty acids (UFA). SFA, such as stearic acid, are present in products such as meat, dairy products, cookies, and pastries. Monounsaturated fatty acids (MUFA) are most frequently consumed via olive oil. The principal series of polyunsaturated fatty acids (PUFA) are n-6 (i.e., linoleic acid) and n-3 (i.e., $\alpha$-linolenic acid, docosahexaenoic acid [DHA], and eicosapentaenoic acid [EPA]). In our Mediterranean dietary pattern, the main sources of n-6 PUFA are vegetable oils, while the principal sources of n-3 PUFA are fatty fish (salmon, tuna, and mackerel). In fact, olive oil contains 70–80% MUFA (oleic acid) and 8–10% PUFA (6–7% linoleic acid and 1–2% $\alpha$-linolenic acid)[16]. The aim of this article was to examine the possible role of dietary fatty acids in modulating the risk of age-related changes in cognitive function and predementia syndromes, as well as the possible mechanisms behind the observed associations.

Furthermore, we reviewed current evidence on dietary fatty acid supplementation in predementia and dementia syndromes.

## Dietary Fatty Acids in ARCD and Predementia Syndromes: Cross-Sectional Studies

At present, an increasing number of epidemiological and clinical studies have addressed the link between UFA intake and cognitive function, most being cross-sectional[16]. In the last years, the study approach was to associate single micro- or macronutrients to ARCD, MCI, AD, or VaD. In this picture, several hallmarks of the Mediterranean diet were linked to increased risk or with a protective effect against cognitive impairment[17]. The typical dietary pattern of the Mediterranean diet is characterized by high intakes of vegetables, fruits and nuts, legumes, cereals, fish, and MUFA; relatively low intakes of meat, and dairy products; and moderate consumption of alcohol. In fact, higher levels of consumption of olive oil are considered the hallmark of the traditional Mediterranean diet.

In a cross-sectional French study on 441 free-living elderly subjects aged 65 or over, a positive relationship was found in women between lipid intake and the Mini-Mental State Examination (MMSE) score, which evaluates global cognitive functions. A positive relationship was also found between PUFA intake and mobility in men, and between functional variables and alcohol intake in the whole sample. The response rate of this study was very low (around 50%) and these findings, contradictory to the results of the subsequent studies, were explained by the authors with the fact that high intakes of these dietary factors can be considered as an indicator of a better health status[18] (Table 1, see pp. 10–15). Another cross-sectional study from Spain investigated the association between dietary intake and global cognitive functions, assessed by the MMSE and the Pfeiffer's Mental State Questionnaire (PMSQ), among 260 noninstitutionalized and nondemented older subjects, aged 65–90 years. The subjects with a lower intake of MUFA, SFA, and cholesterol, and higher intakes of total calories, fresh fruit, carbohydrate, thiamine, foliate, vitamin C, and minerals (iron and zinc), had the best performance in cognitive tests (MMSE score >28 points or no errors on the PMSQ), with a statistical significance after adjustment for age and sex[19] (Table 1).

As seen above, MUFA, consequently to the high consumption of extra-virgin olive oil, represent the most important fats in the Mediterranean diet. Cumulative evidence suggests that extra-virgin olive oil may have a role in the protection against cognitive decline, other than against coronary disease and several types of cancer because of its high levels of MUFA and polyphenolic compounds. The cross-sectional association between dietary macronutrients and cognitive impairment was examined in 278 nondemented elderly subjects, aged 65–84 years, from the Italian Longitudinal Study on Aging (ILSA). A large, population-based, prospective study, with a sample of 5,632 subjects, 65–84 years old, independent or institutionalized, were randomly selected from the electoral rolls of eight Italian municipalities after stratification for age and gender. After adjustment for educational level, the odds ratios (ORs) of cognitive decline (MMSE score <24) decreased exponentially with the increase of MUFA energy intakes. Despite the lower education (≤3 years), MUFA energy intake over 2,400 kJ/day was associated with a reduction in OR of cognitive impairment. The age as a confounder of the interaction term "education by MUFA" was associated with a further increase in OR of cognitive impairment. Furthermore, selective attention performances were independently associated with MUFA intake[20] (Table 1). Recently, another Northern Italian cross-sectional study examined whether type of dietary fats consumed was associated with cognitive performance in a sample of 191 subjects, aged 65 years and older, randomly selected from a free-living population from the Progetto Veneto Anziani (Pro.V.A. study)[21] (Table 1). Global cognitive functions were assessed with the MMSE, comparing subjects with a score between 10 and 17 with subjects with a score between 28 and 30, and plasma phospholipid fatty acid composition was determined by using gas chromatography. In the Pro.V.A. study , in a multiple regression analysis, age and educational level accounted for 29.6% of the MMSE variance, while the contribution of the other variables considered (low-density lipoproteins [LDL] cholesterol, diastolic blood pressure, MUFA, and

PUFA) was almost negligible[21]. The authors acknowledged that these results were limited by the fact that total energy intake, which is known to be reduced in patients with cognitive impairment, was not considered, and by the fact that the study was a cross-sectional survey. Recently, in the Doetinchem Cohort Study, after adjusting for age, gender, education, alcohol consumption, smoking, and energy intake, higher dietary cholesterol was associated with an increased risk of impaired memory function and cognitive flexibility, whereas higher SFA intake was associated with an increased risk of impairment in memory function, psychomotor speed, and cognitive flexibility by 15–19%, although not significantly. Fatty fish and marine n-3 PUFA consumption were significantly associated with a decreased risk of global cognitive function impairment and psychomotor speed by 19–28%. These associations appeared to be independent of differences in cardiovascular risk factors[22] (Table 1). Moreover, Conquer and colleagues measured the plasma fatty acid composition of various phospholipids in blood samples from 84 subjects with different degrees of cognitive impairment, including AD and other types of dementia. Without considering confounding factors, this study showed a statistically significant lower level of n-3 PUFA in the plasma of subjects with cognitive impairment[23].

## Dietary Fatty Acids in ARCD and Predementia Syndromes: Longitudinal Studies

To our knowledge, there is an increasing number of longitudinal epidemiological studies on the association between fatty acids and cognitive functioning[16,24,25,26,27,28,29,30,31], indicating a crucial need for prospective studies that could confirm initial observations. In particular, one of these prospective studies, the Zutphen Study of 476 men aged 69–89 years, found that high linoleic acid intake was positively associated with cognitive impairment in elderly subjects only in cross-sectional study after an adjustment for age, education, cigarette smoking, alcohol consumption, and energy intake. High fish consumption tended to be inversely associated with cognitive impairment and cognitive decline at 3-year follow-up, but not significantly[24] (Table 1). Furthermore, in the cohort of the Etude du Viellissement Arteriel (EVA) Study, moderate cognitive decline (a >2-point decrease on the MMSE) and erythrocyte membrane fatty acid composition were evaluated in 246 elderly subjects aged 63–74 years, during a 4-year follow-up. In this study, a lower content of n-3 PUFA was significantly associated with a higher risk of cognitive decline. After adjusting for age, gender, educational level, and initial MMSE score, stearic acid and total n-6 PUFA were consistently associated with an increased risk of cognitive decline. Moreover, a lower content of n-3 PUFA was significantly associated with cognitive decline, but after adjustment, this association remained significant only for DHA and not for EPA[25] (Table 1). Findings from the Chicago Health and Aging Project (CHAP), on 2,560 persons aged 65 years and older, showed that in a large population-based sample, a high intake of saturated and *trans*-unsaturated fat was associated with a greater cognitive decline over a 6-year follow-up. Intake of MUFA was inversely associated with cognitive change among persons with good cognitive function at baseline and among those with stable long-term consumption of margarine, a major food source. Slower decline in cognitive function was associated with higher intake of PUFA, but the association appeared to be due largely to its high content of vitamin E, which shares vegetable oil as a primary food source and which is inversely related to cognitive decline. Finally, cognitive change was not associated with intakes of total fat, animal fat, vegetable fat, or cholesterol[26] (Table 1). In the same CHAP sample, on 3,718 persons aged 65 years and older, high copper intake was associated with a significantly faster rate of cognitive decline, but only among persons who also consumed a diet that was high in saturated and *trans* fats in a 6-year follow-up[27] (Table 1). Moreover, in a total of 732 men and women, 60 years or older, participating in the EPIC-Greece cohort (European Prospective Investigation into Cancer and Nutrition) and residing in the Attica region, six to 13 years later, seed oil consumption may have adversely affected cognition, whereas adherence to the Mediterranean diet, as well as intake of olive oil, MUFA, and SFA exhibited weakly positive, but not significant associations[28] (Table 1). Finally, 4,809 elderly women (born between 1925 and 1930) were studied in a French longitudinal cohort, the Etude Epidémiologique de Femmes de la Mutuelle Générale de l'Education Nationale (E3N) study. Elderly women participating in the E3N cohort that were reported by informants to have undergone recent cognitive decline had, 13 years previously, lower intakes of poultry, fish, and animal fats, as well as higher intakes of dairy desserts and ice cream. They had lower habitual intakes of dietary fiber and n-3 PUFA, but a higher intake of retinol. Furthermore, elderly women that were reported by informants to be functionally impaired had, in the past, lower intakes of vegetables and vitamins B2, B6, and B12[29] (Table 1).

Therefore, on the basis of the previous significant suggestions[16], we tested further the hypothesis that high MUFA and PUFA intakes may protect against the development of cognitive impairment over time in a median follow-up of 8.5 years of the ILSA. The major finding of this study was that high MUFA, PUFA, and total energy intake were significantly associated with a better cognitive performance in time. Total energy intake should be considered an important confounder of diet-ARCD relationships and, as we proposed in our methodological approach, suggest that association between macronutrient intake and cognitive decline should be adjusted by total energy intake. No other individual dietary components of our study population was significantly associated with cognitive impairment in time[30] (Table 1). The association between high MUFA and PUFA intakes and cognitive performance remained robust even after adjustment for potential confounding variables, such as age, sex, educational level, Charlson Comorbidity Index, body mass index, and total energy intakes[30]. Finally, recent findings from the ILSA demonstrated that while dietary fatty acid intakes were not associated with incident MCI, high PUFA intake appeared to have a borderline nonsignificant trend for a protective effect against the development of MCI[31] (Table 1).

## Fish Consumption in ARCD and Predementia Syndromes

Epidemiological observational studies reporting associations of fish consumption with cognitive function have shown mixed results; some cross-sectional and longitudinal studies have reported a positive association with higher fish consumption[32,33,34,35], while others have found no association[36,37] (Table 1). Fish, particularly fatty fish (e.g., herring, mackerel, salmon, or trout), is the principal source of n-3 PUFA in the Mediterranean diet. Very recently, the baseline data from the Older People And n-3 Long-chain polyunsaturated fatty acid (OPAL) study, a double-blind, randomized, placebo-controlled trial examining the effect of daily supplementation with 700 mg n-3 PUFA (500 mg DHA and 200 mg EPA) for 24 months on cognitive performance in healthy older persons aged 70–79, suggested that higher fish consumption is associated with better cognitive function in later life[32]. Of particular interest was the apparent linear trend for increased cognitive function across the five-item fish consumption variable, with highest cognitive function levels found in those individuals who report eating the largest amount of fatty, as opposed to white, fish[32] (Table 1). In the CHAP, the large population-based sample of 3,718 persons aged 65 years and older, dietary intake of fish was inversely associated with cognitive decline over 6 years of follow-up. In this cohort, there was little evidence that the n-3 PUFA were associated with cognitive change[33] (Table 1). Furthermore, in 210 participants in the Zutphen Elderly Study aged 70–89 years, fish consumers had significantly less 5-year subsequent cognitive decline than did nonconsumers. A linear trend was observed for the relation between the intake of EPA + DHA and cognitive decline, and an average difference of 380 mg/day in EPA + DHA intake was associated with a 1.1-point difference in cognitive decline[34] (Table 1). Finally, findings from the Hordaland Health Study, 2,031 subjects aged 70–74 years from the general population in Western Norway, suggested that subjects whose mean daily intake of fish and fish products was >10 g/day had significantly better mean test scores and a lower prevalence of poor cognitive performance than did those whose intake was <10 g/day. The associations between total intake of seafood and cognition were strongly dose dependent; the maximum effect was observed at an intake of 75 g/day. The effect was more pronounced for nonprocessed lean fish and fatty fish[35] (Table 1).

## DIETARY FATTY ACID SUPPLEMENTATION IN PREDEMENTIA SYNDROMES: IS IT THE CASE FOR A TREATMENT?

The increasing epidemiological evidence of an association between a reduced risk of AD and a diet high in n-3 PUFA and fish consumption[17] is further supported by recent findings that certain diets have been associated with a lower incidence of predementia syndromes[30,31]. One randomized clinical trial (RCT), using an n-3/n-6 fatty acid compound for 4 weeks in 100 AD patients (60 received the fatty acid compound and 40 a placebo control), found improvements in mood, cooperation, appetite, sleep, ability to navigate in the home, and short-term memory[37]. Furthermore, another RCT assessed the effect of supplementation with DHA on cognitive function among 20 elderly nursing home residents with VaD. Hasegawa's Dementia Scale - Revised (HDS-R) and MMSE scores improved in the DHA-treated group, but not among patients who were not treated with DHA. Comparisons between groups were significant at 3 and 6 months for the HDS-R and at 6 months for the MMSE[38].

Recently, Freund-Levi and colleagues examined the effects of dietary n-3 PUFA supplementation, randomizing 204 patients with moderate AD to receive DHA and EPA (for a total dose of 1,720 mg DHA/600 mg EPA) or placebo for 6 months (OmegAD Study). After the treatment period, all the subjects received open-label n-3 PUFA for another 6 months. The authors found that the supplementation did not delay the rate of cognitive decline but, in the group of 32 patients with the most mild AD (MMSE >27, Clinical Dementia Rating Score 0.5-1), n-3 PUFA supplementation slowed the decline in MMSE scores[39]. In addition, the subjects in the placebo group of these very mild AD patients also showed a statistically significant slowing of decline when they were switched to treatment between 6 and 12 months, suggesting that n-3 PUFA might be of benefit to slow the progression of the disease in MCI or very mild AD[39]. Furthermore, this supplementation did not result in marked effects on neuropsychiatric symptoms in mild to moderate AD patients, except for possible positive effects on depressive symptoms and agitation symptoms in subgroups[40]. In fact, there were positive effects on depressive symptoms in non-APOE (non–apolipoprotein E) ε4 carriers and in non-APOE ε4 carriers on agitation symptoms[40].

At present, the effect of arachidonic acid and DHA (240 mg/day) after a 90-day supplementation on MCI, organic brain lesions, or AD showed a significant improvement of the immediate memory and attention score for MCI patients, and a significant improvement of immediate and delayed memories for patients with organic brain damages[41]. The AD group showed no improvement after the supplementation of arachidonic acid and DHA, and the placebo group showed no significant improvement of cognitive functions by the supplementation of 240 mg/day of olive oil (high MUFA content)[41]. The lack of cognitive effects of the olive oil supplementation may probably be explained by the very small amount of olive oil administered in comparison with our ILSA sample in which the mean consumption of olive oil was particularly high: 46 g/day (12.6–113.1 g/day)[20]. Finally, the preliminary results from a 24-week, randomized, double-blind, placebo-controlled study on 23 participants with mild or moderate AD and 23 with MCI randomized to receive n-3 PUFA 1.8 g/day or placebo (olive oil), suggested that n-3 PUFA monotherapy was well tolerable for most of the participants with AD or MCI[42]. This supplementation may improve global clinical function, as measured by the Clinician's Interview-Based Impression of Change scale, which included caregiver-supplied information (CIBIC-plus), relative to placebo. No associations were found between the randomization group and Alzheimer's Disease Assessment Scale – cognitive (ADAS-cog), MMSE, or Hamilton Depression Rating Scale scores. Levels of EPA on erythrocyte membrane were associated with cognitive function, measured by ADAS-cog, in these patients[42]. However, in a secondary analysis, participants with MCI showed more improvement of ADAS-cog than those with AD associated with n-3 PUFA administration[42], supporting recent reports in which PUFA supplementation could be more effective on cognition in people with very mild AD[39] or MCI[41].

In 2006, a Cochrane review team was unable to locate a single published RCT on which to base recommendations for the use of dietary or supplemental n-3 PUFA for the prevention of cognitive impairment or dementia[43]. However, very recently, in a randomized, double-blind, placebo-controlled trial on 302 cognitively healthy (MMSE score >21) individuals aged 65 years or older, the possible impact of n-3 PUFA on the mental well-being and cognitive performance of nondepressed (Center for Epidemiologic Studies Depression Scale score <16), older individuals was investigated[44,45]. In this RCT, participants were randomly assigned to 1,800 mg/d EPA-DHA, 400 mg/day EPA-DHA, or placebo capsules for 26 weeks[44,45]. In older Dutch subjects, no effect of daily supplementation with low or high doses of EPA-DHA on mental well-being as assessed by depression and anxiety questionnaires was found[44]. Furthermore, there were no significant differential changes in any of the cognitive domains (attention, sensorimotor speed, memory, and executive function) for either low- or high-dose fish oil supplementation compared with placebo[45]. However, an effect of EPA-DHA supplementation in subjects who carried the APOE ε4 allele was found, but only on the cognitive domain of attention[45]. Fish oil may be beneficial in those subjects who are most sensitive to developing dementia. These two substantially negative studies on ARCD may be explained by the samples investigated (nondepressed and noncognitively impaired older subjects). Further trials in depressed patients or ε4 carriers with MCI are needed. Finally, there is another ongoing RCT with cognitive end points of n-3 PUFA supplementation in healthy, cognitively intact, older persons. The OPAL study examined healthy older persons aged 70–79 with good cognitive function (MMSE ≥24 out of 30 points at baseline) who are recruited from 20 primary care practices[46].The OPAL study was completed at the end of 2007 and findings will be published shortly. Therefore, epidemiological evidence suggested a possible association between PUFA (particularly, n-3 PUFA) and reduced risk of cognitive decline and dementia. However, due to the small number of studies that inform about this topic, further research is necessary before a strong conclusion can be drawn. Some recent RCTs assessed the cognitive or functional effect of n-3 PUFA supplementation on patients with VaD, AD, MCI, or ARCD in cognitively unimpaired older subjects. These RCTs suggested a positive effect of this intervention only in very mild AD or MCI patients, or in subgroups (e.g., APOE e4 carriers) for cognitive performance in nondemented subjects or for neuropsychiatric symptoms in mild to moderate AD patients. Supplemental DHA may not necessarily be recommended when the decline is more severe, possibly because additional DHA might contribute to degenerative processes in the brain related to lipid peroxidation. Furthermore, the breadth of doses of DHA used in these studies covered a 36-fold range (from 120 mg/day to 4.3 g/day), a huge range that, importantly, produced no apparent dose-related effects on cognition. On the basis of these evidences, we also strongly suggest for predementia syndromes, a high-risk condition for progression to dementia of vascular and degenerative origin, intervention trials using measures of dietary supplementation similar to the OmegAD Study in order to determine if such supplements will slow cognitive decline[47].

## FATTY ACIDS AND COGNITIVE DECLINE: POSSIBLE MECHANISMS

Different pathways could contribute to the neuroprotective as well as the neurotrophic properties of UFA[48,49]. In the older subjects of the ILSA, which fulfilled a Mediterranean dietary pattern, total fat is 29% of energy, with a high consumption of olive oil (46 g/day), a MUFA energy intake of 17.6% of total energy, 85% of which derived from olive oil, and a SFA intake of only 6%[20]. In our population, the prolonged protection of MUFA intake against age-related changes in cognitive functions may be linked to the relevant quota of antioxidant compounds in olive oil, including low molecular weight phenols[20]. In fact, animal studies suggested that diets high in antioxidant-rich foods, such as spinach, strawberries, and blueberries, rich in anthocyanins and other flavonoids, may be beneficial in slowing ARCD[50]. The possible role of antioxidant compounds from olive oil do not diminish or otherwise alter the argument concerning the fatty acids, because this is only a possible explanation of the role of MUFA on age-related cognitive changes in our population, in which MUFA intake derived for a large part from olive oil.

In adult rats, learning and cognitive behavior are related to brain DHA status, which, in turn, is related to the levels of the dietary n-3 PUFA[51]. In fact, administration of DHA seems to improve learning ability in β-amyloid (Aβ)–infused rats[52] and inhibit decline in avoidance-learning ability in the AD model rats, associated with an increase in the cortico-hippocampal n-3/n-6 ratio, and a decrease in neuronal apoptotic products[53]. Similarly, recent studies showed that dietary DHA in an aged AD mouse model could be protective against Aβ production, deposition in plaques and cerebral amyloid angiopathy[54,55], and increases cerebral blood volume[55]. In other transgenic AD mouse models, DHA also protects against dendritic pathology[56] and prevents neuronal apoptosis induced by soluble Aβ peptides[57], increases synaptic protein and phospholipid densities[58,59], and inhibits degradative endopeptidase activities[60]. Some experimental evidence suggested that essential n-3 PUFA effectively lower Aβ production in transgenic mice, as reported in studies from several laboratories[54,58,61]. Yet, plaque burden was reduced in one study using aged transgenic mice following a 3-month DHA-enriched diet[54], but not in other studies that started dietary intervention at a much younger age[55,61].

The neuroprotective effects of dietary UFA could rely on their impact on membrane architecture. In fact, UFA have an important role in maintaining the structural integrity of neuronal membranes, determining the fluidity of synaptosomal membranes, and thereby regulating neuronal transmission. Furthermore, essential fatty acids can modify the activity of certain membrane-bound enzymes (phospholipase A2, protein kinase C, and acetyltranferase) and the function of the neurotransmitters' receptors. Finally, free fatty acids, lipid metabolites, and phospholipids modify the function of membrane proteins, including ion channels[62]. Moreover, fatty acid composition of neuronal membranes in advancing age demonstrated an increase in MUFA content and a decrease in PUFA content[63]. n-3 PUFA increase membrane fluidity by replacing n-6 PUFA and cholesterol from the membrane[64], maintaining an optimal membrane fluidity as obligatory for neurotransmitter binding and signaling within the cell[65]. There is also evidence that associates a dietary deficiency of n-3 PUFA with changes in cortical dopaminergic function[66]. The n-3 PUFA from fish may be inversely associated with dementia because it lowers the risk of thrombosis[67], stroke[68], cardiovascular disease[69], and cardiac arrhythmia, reducing the risk of thromboembolism in the brain and consequently of lacunar and large infarcts that can lead to VaD and AD. Furthermore, the n-3 PUFA may be as important as lipids in the brain, particularly for the possible influence of DHA on the physical properties of the brain that are essential for its function[70]. Furthermore, fish oil was a better source than α-linolenic acid for the incorporation of n-3 PUFA into rat brain phospholipid subclasses[62]. Conversely, high linoleic acid intake (n-6 PUFA) may increase the susceptibility of LDL cholesterol to oxidation, which makes it more atherogenic[71], even if the association between linoleic acid and atherosclerosis is controversial[72]. Therefore, the ratio of dietary n-3/n-6 PUFA intake may influence the potential role of PUFA on cognitive decline and dementia, the optimal ratio of n-6/n-3 for an healthier diet should be <5:1[73]. Finally, a high dietary intake of SFA and cholesterol increases the risk for cardiovascular disease, and therefore for cognitive decline, VaD, and AD[13]. Conversely, treatment for 4 weeks with a Mediterranean-inspired diet rich in n-3 PUFA decreased blood lipids in healthy individuals with a low-risk profile for cardiovascular disease, with a beneficial effect also on vascular function and oxidative stress[74].

## CONCLUSIONS

At present, several studies suggest that an increase of SFA could have negative effects on cognitive functions. Furthermore, a clear reduction of risk for cognitive decline has been found in population samples with elevated fish consumption, and high intake of MUFA and PUFA, particularly n-3 PUFA. Recent findings demonstrated that while dietary fatty acid intakes were not associated with incident MCI, high PUFA intake appeared to have a borderline nonsignificant trend for a protective effect against the development of MCI. Nonetheless, at present, no definitive dietary recommendations on fish and UFA consumption, or lower intake of saturated fat, in relation to the risk for dementia and cognitive decline are possible. In fact, in a recent RCT, n-3 PUFA supplementation did not influence cognitive functioning during a follow-up of 6 months, except in a small group of patients with very mild AD and for possible positive effects on depressive symptoms in non-APOE ε4 carriers. These data, together with epidemiological evidence, support the idea that n-3 PUFA may have a role in the primary and maybe secondary prevention of the disease, but not when the disease process has already taken over[75]. However, high levels of consumption of fats from fish, vegetable oils, and vegetables should be encouraged because this dietary advice is in accordance with recommendations for lowering the risk of cardiovascular disease, obesity, diabetes, and hypertension. Therefore, epidemiological studies on the association between diet and cognitive decline suggested a possible role of fatty acid intake in maintaining adequate cognitive functioning, and possibly in preventing or delaying the onset of dementia, both of degenerative or vascular origin. Appropriate dietary measures or supplementation with specific macronutrients might open new ways for the prevention and management of cognitive decline and dementia[76].

On the other hand, Scarmeas and colleagues, using the inclusive dietary score (MeDi score), but studying a population with a substantial difference in dietary habits in comparison with Greek (EPIC)[28] and Italian (ILSA)[30,31] cohorts, found that higher adherence to the Mediterranean diet is associated with a trend for reduced risk of incident MCI and with reduced risk of MCI progression to AD[77]. The use of diet scoring systems, such as the MeDi score[78], has undeniable advantages in furthering the understanding of the role of diet in chronic disease[79]. They may account for the complex biologic interactions between different components of a composite dietary pattern, such as Mediterranean diet, that may be difficult to detect in analyses focusing only on the individual components[80]. These contrasting findings about the impact of the MeDi score or individual macronutrients on ARCD or MCI may suggest an approach not confined only to cognitive skills, but extended to functional status and comorbidity. However, we should not renounce *a priori* the work for a correct estimate of the validity of the MeDi score for cognitive impairment as a criterion. In fact, the evidence about the role of the whole Mediterranean diet on cognitive decline is still scarce[33,77]. Therefore, in future studies, it could also be indicated, together with measures of this effect by a dietary composite score, that the estimates and the impact of the individual components of the diet be reported. In a very recent reanalysis from the ILSA cohort, we showed that high PUFA were associated with reduced risk of incident MCI among those who consumed a low MUFA/SFA ratio intake[81]. In fact, while an increasing body of evidence suggested that elevated fish consumption and high intake of n-3 PUFA may be protective against ARCD and MCI, the traditional Cretan diet, although strongly dependent on high olive oil intake, was never centered on fish consumption[79]. In this context, n-6 PUFA could potentially exert some health benefits. In fact, in the Mediterranean diet, some foods are rich in n-6 PUFA (e.g., walnuts, almonds, hazelnuts), while other foods, although poor in n-6 PUFA, are highly consumed, such as cereals, legumes, and, in less amounts, some types of meat (pork) and poultry. Furthermore, olive oil contains n-6 and n-3 PUFAs in a ratio of 10:1. Therefore, it should be advisable to include PUFA in the MeDi score as individual macronutrients (such as MUFA/SFA ratio), among the components presumed to be beneficial, in evaluating the relationship between adherence to the Mediterranean diet and ARCD or MCI[81].

## ACKNOWLEDGMENTS

This work was supported by the Italian Longitudinal Study on Aging (ILSA) (Italian National Research Council - CNR-Targeted Project on Ageing - Grants 9400419PF40 and 95973PF40) (Dr. Solfrizzi Dr. C. Capurso, Dr. D'Introno, Dr. Colacicco, Pr. A. Capurso, and Dr. Panza). The authors thank Ms. Maria Mann for editing the manuscript.

**TABLE 1**
**Principal Cross-Sectional and Longitudinal Clinical and Epidemiological Studies on the Relationships between Dietary Fatty Acids or Fish Consumption and Predementia Syndromes (i.e., ARCD and MCI) in Older People**

| Ref. | Setting and Study Design | Subjects | Dietary Assessment | Cognitive Outcomes | Results and Conclusions | Comment |
|---|---|---|---|---|---|---|
| **Cross-sectional studies** | | | | | | |
| Pradignac et al.[18] | Cross-sectional, population-based | 441 subjects, aged 65 years and older | Evaluation of dietary intake | MMSE, Geronte scale for the assessment of daily living activities | In men, alcohol intake was associated with improved functional and cognitive parameters, while PUFA intake only with functional status. In women, lipid intakes were related to better cognitive performance. Overweight in both sexes was associated with an improvement in functional status. | One of the first studies on the possible role of macronutrients in ARCD. The response rate of this study was very low (around 50%) and these findings, contradictory to the results of the subsequent studies, were explained by the authors with the fact that high intakes of these dietary factors can be considered as an indicator of a better health status. |
| Ortega et al.[19] | Cross-sectional | 260 subjects, aged 65–90 years | Evaluation of dietary intake with a weighed-food record for 7 consecutive days, and biochemical assays | MMSE, PMSQ | A diet poor in MUFA, SFA, and cholesterol, but rich in carbohydrates, fibers, vitamins (folates, vitamins C and E, and β-carotene, and minerals [zinc and iron]) seems to improve cognitive skills. | The apparently conflicting findings on MUFA intake and cognitive function between this study and others could be partially due to some methodological differences in FFQs and selection of participants. |
| Solfrizzi et al.[20] | Cross-sectional, population-based | 278 subjects, 65–84 years old | Evaluation of dietary intake with a 77-item FFQ | MMSE, DCT, and BSRT | Inverse relationship between MUFA intake and cognitive decline. Significant inverse association between MUFA intakes and selective attention. No association was found between nutritional variables and episodic memory. | This study suggested that high MUFA intakes appeared to be protective against ARCD, with the obvious limitations of a cross-sectional survey with a limited number of subjects. |

*Table 1 continues*

**TABLE 1 (continued)**

| Ref. | Setting and Study Design | Subjects | Dietary Assessment | Cognitive Outcomes | Results and Conclusions | Comment |
|---|---|---|---|---|---|---|
| Manzato et al.[21] | Cross-sectional, population-based | 191 subjects, aged 65 years and older | Evaluation of plasma phospholipid fatty acid composition | MMSE: subjects with a score between 10 and 17 vs. subjects with a score between 28 and 30 | Cognitive functioning is affected mainly by age and education, not by dietary fatty acids. | The authors acknowledged that these results were limited by the fact that total energy intake, which is known to be reduced in patients with cognitive impairment, was not considered, and by the fact that the study was a cross-sectional survey. |
| Kalmijn et al.[22] | Cross-sectional, population-based | 1,613 subjects, 45–70 years old | Evaluation of fatty fish, total fat, cholesterol, SFA, MUFA, PUFA (n-6 and n-3) dietary intakes with a 178-item FFQ | Concurrent to the dietary assessment, the VVLT, the CST, an abbreviated SCWT, the LDST, a CFT were administered | Fatty fish and marine n-3 PUFA consumption was associated with a reduced risk and intake of cholesterol, and SFA with an increased risk of impaired cognitive function in this middle-aged population. | The association between SFA and cognitive function appeared to be independent of differences in cardiovascular risk factors. |
| Nurk et al.[35] | Cross-sectional, population-based | 2,031 subjects, 70–74 years old | Evaluation of dietary intakes with a 169-item FFQ | Six cognitive tests were administered: m-MMSE, m-DST, m-BD, KOLT, TMT-A, and the S-task of the COWAT | Consumers of fish and fish products had better cognitive function than did nonconsumers. The associations between fish and fish product intake and cognition were dose dependent. The effect of fish on cognition differed according to the type of fish and fish product consumed. | Although of cross-sectional design, this study had a large population-based sample with a relatively high consumption of fish and fish products, and with interesting dose-response analyses. |

Table 1 continues

**TABLE 1 (continued)**

| Ref. | Setting and Study Design | Subjects | Dietary Assessment | Cognitive Outcomes | Results and Conclusions | Comment |
|---|---|---|---|---|---|---|
| Dangour et al.[32] | Randomized clinical trial (24 months) | 867 subjects, 70–79 years old, from 20 general practices in England and Wales | Evaluation of fish consumption variable that took into account both frequency and type of fish consumed | A standardized battery of cognitive tests: CVLT; subjective memory assessment; three tests of prospective memory; story recall (immediate and delayed); verbal fluency; letter cancellation; location memory (immediate and delayed); symbol-letter substitution; digit span forwards and backwards; simple and choice reaction time | High levels of fish consumption are associated with better cognitive function in later life. | Of particular interest, although in a cross-sectional study, there was an apparent linear trend for increased cognitive function across the five-item fish consumption variable, with highest cognitive function levels found in those individuals who report eating the largest amount of fatty, as opposed to white. fish. |
| **Longitudinal studies** | | | | | | |
| Kalmijn et al.[24] | Longitudinal, population-based (3 years) | 476 subjects, aged 69–89 years | Evaluation of dietary intake with the cross-check dietary history method | Cognitive impairment defined as a MMSE score <25 points and cognitive decline as a drop of >2 points of MMSE over a 3-year period | High linoleic acid intake (PUFA) was positively associated with cognitive impairment. High fish consumption was inversely associated with cognitive impairment. | The first longitudinal study on the possible role of dietary fatty acids and fish consumption on ARCD. |
| Hende et al.[25] | Longitudinal, population-based (4 years) | 246 subjects, aged 63–74 years | Evaluation of fatty acid composition in erythrocyte membranes | MMSE score with a >2-point decrease in a 4-year follow-up | Inverse association between cognitive decline and the ratio of n-3 to n-6 PUFA in erythrocyte membranes | This is the first report relating the fatty acid composition of erythrocyte membranes to cognitive decline in the elderly. No dietary intake data were collected in this study, but the erythrocyte membrane fatty acid composition can reflect dietary fat intake. |

*Table 1 continues*

**TABLE 1 (continued)**

| Ref. | Setting and Study Design | Subjects | Dietary Assessment | Cognitive Outcomes | Results and Conclusions | Comment |
|---|---|---|---|---|---|---|
| Morris et al.[26] | Longitudinal, population-based (6 years) | 2,560 subjects, aged 65 years and older | Evaluation of dietary intake with a 139-item FFQ | Cognitive change at 3- and 6-year follow-ups measured with the EBT of Immediate and Delayed Recall, the MMSE, and the SDMT | A diet high in saturated and *trans*-unsaturated fat, or low in nonhydrogenated unsaturated fats may be associated with cognitive decline among older people. | The first longitudinal study in which higher SFA intake was associated with cognitive decline, and higher MUFA intake and PUFA/SFA protected against ARCD. |
| Morris et al.[33] | Longitudinal, population-based (6 years) | 3,718 subjects, aged 65 years and older | Evaluation of dietary intake with a 139-item FFQ | Cognitive change at 3- and 6-year follow-ups measured with the EBT of Immediate and Delayed Recall, the MMSE, and the SDMT | Dietary intake of fish was inversely associated with cognitive decline over 6 years. There were no consistent associations with the n-3 fatty acids, although the effect estimates were in the direction of slower decline. | Longitudinal study confirming the protection of fish consumption, but not of higher intake of n-3 PUFA against ARCD. |
| Morris et al.[27] | Longitudinal, population-based (6 years) | 3,718 subjects, aged 65 years and older | Evaluation of dietary intake with a 139-item FFQ | Cognitive change at 3- and 6-year follow-ups measured with the EBT of Immediate and Delayed Recall, the MMSE, and the SDMT | High copper intake was associated with a significantly faster rate of cognitive decline, but only among persons who also consumed a diet that was high in saturated and trans fats. | Study suggesting an accelerated cognitive decline among persons whose diets were high in copper and saturated and *trans* fats, confirming some suggestions of previous case-control studies with elevated serum copper in AD patients. |
| Solfrizzi et al.[30] | Longitudinal, population-based (8.5 years) | 278 subjects, 65–84 years old, from a cohort of 5,632 subjects | Evaluation of MUFA and PUFA dietary intakes with a 77-item FFQ | MMSE | High MUFA, PUFA, and total energy intake were significantly associated with a better cognitive performance in time. The association between high MUFA and PUFA intakes and cognitive performance remained robust even after adjustment for potential confounding variables, such as age, sex, educational level, CCI, BMI, and total energy intakes. | Longitudinal confirmatory study on the protective role of MUFA and PUFA against ARCD with a longer follow-up period. |

*Table 1 continues*

**TABLE 1 (continued)**

| Ref. | Setting and Study Design | Subjects | Dietary Assessment | Cognitive Outcomes | Results and Conclusions | Comment |
|---|---|---|---|---|---|---|
| Solfrizzi et al.[31] | Longitudinal, population-based (2.6 years) | 278 subjects, 65–84 years old, from a cohort of 5,632 subjects | Evaluation of MUFA and PUFA dietary intakes with a 77-item FFQ | Incident MCI. Diagnostic criteria for MCI: 1.5 S.D. below mean age- and education-adjusted on the MMSE and 10th percentile below age- and education-adjusted on memory test, without SMC and intact ADL/IADL | Dietary fatty acid intakes were not associated with incident MCI. However, high PUFA intake appeared to have a borderline nonsignificant trend for a protective effect against the development of MCI that may be important. | In this population-based study, dietary fatty acid intakes were associated for the first time with incident MCI, although in a limited sample with a brief follow-up period. |
| van Gelder et al.[34] | Longitudinal, population-based (5 years) | 210 subjects, 70–89 years old | Information about habitual food consumption was collected using the cross-check dietary history method | MMSE | Fish consumption was associated with less subsequent 5-year cognitive decline than no fish consumption. Furthermore, a dose-response relation was noted between the combined intake of EPA and DHA and cognitive decline, which suggests that a higher intake of EPA plus DHA was associated with less cognitive decline. | Possible explanations for the discrepancy between the earlier results of the Zutphen Elderly Study[24] and these findings could be the longer follow-up period and the availability of data on the EPA and DHA content of animal and plant foods in addition to fish and seafood. |
| Psaltopoulou et al.[28] | Longitudinal, population-based (median 8 years) | 732 subjects, 60 years or older | Evaluation of dietary intakes with a 150-item FFQ. A dietary composite score (MeDi score) evaluated adherence to Medi-terranean diet. | MMSE | No significant association between MeDi score and MMSE scores, whereas a statistically significant inverse association was found between MMSE performance and some individual dietary components, such as seed oil or PUFA intakes. | Although a longer median follow-up period, the lack of cognitive assessment at baseline did not allow the estimation of patterns of decline, and to document conclusively the causal nature and the directionality of associations. |

*Table 1 continues*

**TABLE 1 (continued)**

| Ref. | Setting and Study Design | Subjects | Dietary Assessment | Cognitive Outcomes | Results and Conclusions | Comment |
|---|---|---|---|---|---|---|
| Vercambre et al.[29] | Longitudinal, population-based (13 years) | 4,809 elderly women, 76–82 years old | Evaluation of dietary intakes with a 208-item FFQ | DECO and IADL | Elderly women that were reported by informants to have undergone recent cognitive decline had, 13 years previously, lower intakes of poultry, fish, and animal fats, as well as higher intakes of dairy desserts and ice cream. They had lower habitual intakes of dietary fiber and n-3 PUFA, but a higher intake of retinol. Furthermore, elderly women that were reported by informants to be functionally impaired had, in the past, lower intakes of vegetables and vitamins B2, B6, and B12. | The main interest of this study lay in the time interval (more than a decade) between dietary assessment and cognitive and functional assessment, although in a sample of only women, so limiting the generalizability of the findings. |

ADL = activities of daily living; BMI = body mass index; BSRT = Babcock Recall Story Test (episodic memory); CCI = Charlson Comorbidity Index; CFT = Category Fluency Test (semantic memory); CST = Concept Shifting Task (mental processing speed); CVLT = Californian Verbal Learning Test (verbal memory); DCT = Digit Cancellation Test (selective attention); DECO = DEtérioration Cognitive Observéè scale (observed cognitive deterioration); DHA = docosahexaenoic acid; EBT = East Boston Memory test (immediate and delayed episodic memory); EPA = eicosapentaenoic acid; FFQ = food frequency questionnaire; IADL = instrumental activities of daily living; KOLT = Kendrick Object Learning Test (episodic memory); LDST = Letter Digit Substitution Test (perceptual-motor speed); m-BD = modified Block Design (visuospatial and motor skills); m-DST = modified Digit Symbol Test (perceptual speed); m-MMSE = modified Mini-Mental State Examination (global cognitive functioning); MMSE = Mini-Mental State Examination (global cognitive functioning); PMSQ = Pfeiffer's Mental State Questionnaire (global cognitive functioning); SCWT = Stroop Color Word Test (selective attention); SDMT = Symbol Digit Modalities Test (perceptual-motor speed); SMC = Subjective Memory Complaint; S-task of the COWAT = the abridged version of the Controlled Oral Word Association Test (access to semantic memory); TMT-A = Trail Making Test, part A (executive function); VVLT = Visual Verbal Learning Test (verbal memory).
